# Magnitude of Gender-Based Violence and Its Associated Factors among Female Night Students in Bahir Dar City, Amhara Region, Ethiopia

**DOI:** 10.1155/2021/6694890

**Published:** 2021-04-12

**Authors:** Habtamu Gebrehana Belay, Tewachew Muche Liyeh, Habtamu Abie Tassew, Abeba Belay Ayalew, Yitayal Ayalew Goshu, Gedefaye Nibret Mihretie

**Affiliations:** Department of Midwifery, College of Health Science, Debre Tabor University, Debre Tabor, Ethiopia

## Abstract

**Background:**

Gender-based violence (GBV) is a major public health issue that affects the health and well-being of millions of young people worldwide each year. Gender-based violence was prevalent throughout Sub-Saharan Africa. However, research in Africa is extremely diverse.

**Objective:**

The purpose of this study is to determine the extent and risk factors of gender-based violence among night female students in Bahir Dar, Ethiopia.

**Methods:**

This cross-sectional study was conducted among 788 elementary and high school night female students in Bahir Dar from October 15 to November 15, 2019. Data was gathered using self-administered questionnaires. A binary and multiple logistic regression model was used to identify factors associated with gender-based and sexual violence. An adjusted odds ratio (AOR) with a 95 percent confidence interval (CI) was calculated to determine the level of significance.

**Results:**

The overall lifetime prevalence of gender-based violence (sexual, physical, and emotional violence) was 71.1% with a 95% CI of 67.8%-74.1%. The lifetime prevalence of sexual violence, physical violence, and emotional violence was 49.1%, 57.5%, and 41.6%, respectively. Rural childhood residence (AOR: 3.37, 95% CI: (2.17-5.54)), low school performance (AOR: 3.44, 95% CI: (2.13-5.56)), lack of sexual and reproductive health conversation experience (AOR: 3.68, 95% CI: (2.07-6.54)), poor family control (AOR: 5.62, 95% CI: (3.25-9.71)), and being sexually active (AOR: 3.79, 95% CI: (2.35-6.12)) increased significantly the risk of gender-based violence. The risk factors for sexual violence were young people living with both parents (AOR: 0.28, 95% CI: (0.19-0.41)), peer pressure (AOR: 5.73, 95% CI: (4.11-7.98)), and family support (AOR: 0.31, 95% CI: (0.22-0.43)).

**Conclusion:**

In the study area, the lifetime prevalence of sexual violence, physical violence, and emotional violence was high. As a result, it is recommended that policymakers, district officials, schools, and other stakeholders pay attention to and act on gender-based values.

## 1. Background

Gender-based violence is defined as violence directed against an individual based on sex or gender, which results in psychological, physical, or sexual trauma, either directly or indirectly [1]. Gender-based violence (GBV) is one of the most widespread, underreported, and unsolicited human rights violations in the world. It has no social, economic, or national boundaries. Violence against women is a major public health problem affecting the physical, sexual, mental, and social well-being of more than one-third of all women worldwide [2]. Violence against women and girls is one of the most common human rights violations in the world. Providing high-quality care and support services to victims of violence is critical for reducing trauma, assisting victims in healing, and preventing repeat victimization and perpetration [3].

Sexual violence affects people of all genders, sexual orientations, and ages in every community. Anyone can be a victim of sexual violence, but the majority of victims are women [4]. Sexual and gender-based violence is a serious, life-threatening global problem that affects women, girls, boys, and men, and it is widely underreported in all settings. All forms of violence are strongly linked to social determinants such as poor governance, weak rule of law, cultural, social, and gender norms, unemployment, income, and gender inequality, and limited educational opportunities [5].

Globally, one in every three women has experienced physical and/or sexual intimate partner violence in her lifetime, and one in every three adolescent girls reports having their first sexual experience as a result of coercion [6, 7]. Globally, it is estimated that up to 1 billion children aged 2–17 years have been victims of physical, sexual, or emotional violence [8]. Gender-based violence is common in Sub-Saharan African educational institutions (SSA). Gender-based violence in schools lowers girls' educational attainment and increases absenteeism and dropout rates. More than 40% of women have experienced some form of intimate partner violence at some point in their lives [9]. The consequences of GBV include murder, suicide, HIV/AIDS, shock, infection, chronic pain, alcohol/drug dependence, miscarriage, unwanted pregnancy, unsafe abortion, victim/survivor guilt, anxiety, fear, self-hatred, and self-denial, economic dependence, loss of role or function in society [10].

The systemic review in Ethiopia has shown that the lifetime prevalence of domestic violence against women by husband or intimate partner violence ranges from 20 to 78%. The lifetime prevalence of domestic physical violence by a husband or intimate partner against women ranged from 31 to 76.5%, and the lifetime prevalence of domestic sexual violence against women by husband or intimate partner ranged from 19.2 to 59% [11]. Previous research on gender-based violence in Ethiopia has focused on daytime high school students, but this study is aimed at determining the magnitude and associated factors of gender-based violence among female primary and high school students at night.

## 2. Methods

### 2.1. Study Design and Setting

This cross-sectoral institutional study was conducted among 788 elementary and high school night students in Bahir Dar City, Ethiopia, from October 15, 2019, to November 15, 2019. Bahir Dar is the capital of the regional state of Amhara. It is located approximately 565 km away from the capital city of Ethiopia, Addis Ababa.

### 2.2. Population

All-female night students registered in public institutions in Bahir Dar City for the academic year 2019 were the source population and those identified in the chosen public institution during the data collection period were the study population.

### 2.3. Inclusion and Exclusion Criteria

All-female night students enrolled in a night program at a public elementary and high school in Bahir Dar were included, while female night students who had lived in the city for less than six months were excluded.

### 2.4. Sample Size Determination

The single population proportion formula was used to determine the sample size using 58.3% prevalence of lifetime gender-based violence among high school students [12]. Assuming 95% confidence interval (CI), 5% margin of error, design effect 2, and additional 10% for nonresponse rate and the final sample size were 823.

### 2.5. Sampling Procedure

Based on the percentage of female students in grades 1-8 and 9-10 in each class, the calculated sample size was proportionally allocated to their respective elementary and high school students. Students' rosters or name lists were used as a sampling frame to select study subjects from each grade. The total of students aging 15-24 years in each grade were divided by the proportionally allocated sample size to each grade to get the interval (K). The first respondent was selected by using lottery methods.

### 2.6. Operational Definition

- *Gender-Based Violence*. Results in sexual, psychological, and physical trauma
- *Sexual Violence*. Unwanted or nonconsensual sexual act through force, threat, or intimidation
- *Sexual Harassment*. Unwanted sexual behaviors including jokes, verbal communications, and intentional physical contacts
- *Physical Violence*. In this study, physical violence is defined as a respondent saying “yes” to one of the following items: slapping, kicking, beating with any object, cutting/biting, shaking, shoving, pushing, throwing, and burning/chocking against an individual

### 2.7. Data Collection Procedure

Data from participants were collected using a pretested interviewer-administered structured questionnaire adapted from literature reviews developed by different authors for similar purposes [13].To ensure the consistency and content of the questionnaire, the questioner was first written in English, then translated into Amharic, and then retranslated back into English. Five Bachelor of Science midwives collected data under the supervision of two Master of Science in clinical midwives.

### 2.8. Data Quality Assurance

To avoid confusion and to ensure a common understanding of the study, data collectors and supervisors were trained regarding the objectives of the study, data collection methods, and significance of the study. A pretest was conducted for 10% of the total sample at another public institution with similar characteristics to the study population. Throughout the data collection process, interviewers were supervised, and regular meetings were held between the data collectors, the supervisor, and the principal investigator to discuss and address any issues that arose during the interviews. Before entering data, the collected data were reviewed and checked for completeness.

### 2.9. Data Analysis

The collected data were checked, coded, and entered into Epi-data (version 3.5) software, then exported into SPSS software (version 23) for analysis. Bivariate analysis was performed for all explanatory variables about gender-based violence and sexual violence. Variables having a *p* value < 0.20 in the bivariate analysis were selected for the multivariate logistic regression modeling, for adjustment of confounding effects between explanatory variables. Adjusted odds ratio (AOR) with 95% CI was computed, and variables having *p* value < 0.05 in the multivariate logistic regression model were considered as statistically significant. The odds ratio was also used to determine the strength of association between independent variables and the outcome variable.

## 3. Results

### 3.1. Sociodemographic Characteristic of Study Participants

A total of 788-night female students were enrolled, with a response rate of 95.75 percent. The mean age of participants in the study was 18.88 years with SD ± 2.73. The majority of respondents 683 (86.7%) were Orthodox religious followers and 718 (91.1%) were Amharic ethnic. More than half of the 463 (58.8%) childhood residences of study participants were urban, and 325 (41.1%) were rural residences. More than two-thirds of participants 514 (65.2%) were grades 1-8, and 274 (34.8%) were from grades 9-10. The current living conditions of the respondents were 118 (15%) alone, 214 (27.2%) with the family, and 245 (31%) with the employer. More than half of 423 (53.7%) were single in marital status. The monthly pocket money of respondents that got less than 2000 Ethiopian birr was 324(41%) and got greater than 4000 Ethiopian birrs was 184(23.4%) (Table 1).

### 3.2. Sexual Experience and Sexual Violence

Of the total respondents, 490 (62.2%%) had sexual experience. More than two-thirds of participants 319 (65.1%) started the first sex at the age of between 17-19 years, and 90 (18.4%) started their first sex at age greater than 20 years and above. Three-hundred and ten 310 (63.3%) participants had more than one sexual partner in their lifetime, and 180 (36.7%) had only one sexual partner in their lifetime. The first exposure for the first sexual intercourse was peer pressure 220 (46.7%), forced 59 (12%), and 50 (10.2%) self-interest. Of the total respondents, less than one-third 227(28.8%) were discussed abort sexual and reproductive issues with family and or friends. About four in ten young people (41.2%) had a constant sexual partner. Of the 788 total respondents, 387 (49.1%) had experienced sexual violence in their lifetime. The attempted sexual harassment reported by two-thirds of the respondents was 514 (65.2%), and the lifetime prevalence of rape was 132 (16.7%) (Table 2). There are several consequences of gender-based violence mentioned by the despondences. Those complications were unwanted pregnancy 72 (14.7%), depression 323 (66%), anxiety 303 (62%), self-blame 216 (44), and injury around the genitalia 201 (41%) (Table 3).

### 3.3. Factors Associated with Gender-Based Violence

Multivariate logistic regression revealed that rural childhood residence, discussing sexual and reproductive health issues with family and/or friends, family control, and being sexually active were all significantly associated with gender-based violence. Living in a rural area during childhood increased the risk of GBV by three times as compared to their counterparts (AOR: 3.37, 95% CI: (2.17-5.54)). Students who had no prior experience discussing sexual and reproductive health matters with family or friends had nearly four times increased the risk of GBV as compared to their counterparts (AOR: 3.68, 95% CI: (2.07-6.54)). Students who performed poorly in school had three times the odds of experiencing GBV as those who performed well in school (AOR: 3.44, 95% CI: (2.13-5.56)). Students who had poor family control were increased almost 4 times the odds of GBV as compared to those who had good family control (AOR: 5.62, 95% CI: (3.25-9.71)) and being sexually active increased almost 4 times the odds of GBV as compared to their counterparts (AOR: 3.79, 95% CI: (2.35-6.12))(Table 4).

Living with a biological partner, peer pressure, and family support was all found to be significantly associated with sexual violence in multivariate logistic regression analyses. Night female students living with their biological partners reduced the risk of gender-based violence by 72% as compared to their counterparts (AOR: 0.28, 95% CI: (0.19-0.41)). Peer pressure increased almost 4 times the odds of GBV (AOR: 5.73, 95% CI: (4.11-7.98)), and family support reduced the risk of gender-based violence by 69% as compared to those who did not get monthly pocket money, lack of psychological support, and physical control (AOR: 0.31, 95% CI: (0.22-0.43)) (Table 5).

## 4. Discussion

The overall lifetime prevalence of gender-based violence was found to be 71.1%. The lifetime prevalence of sexual violence, physical violence, and emotional violence was found to be 49.1%, 57.5%, and 41.6%, respectively. This finding is consistent with studies conducted in Wolaita Sodo town, Debre Markos town, and Aleta Wondo town, which shows that the lifetime prevalence of gender-based violence was 63.2%, 67.7%, and 68.2%, respectively [14–16]. This finding is also supported by studies conducted in different parts of Ethiopia; violence against women was 64.7%; in southwest Ethiopia, the lifetime prevalence of any form of intimates partner violence was 72% in rural Ethiopia, and the prevalence of domestic violence in Fagitalekoma woreda, Awi zone, Amhara regional state was 78% [17–19]. This is might be due to similar sociodemographic characteristics.

This finding is more prevalent than studies conducted in Northern Nigeria, Zimbabwe and Uganda, which revealed that the overall prevalence of gender-based violence was 58.8%, 43.4%, and 47%, respectively [20–22]. Furthermore, this finding is much higher than other different findings; the prevalence of gender-based violence in US women was 25%, 13.9% in South Africa, and 35.6% was among women in Somalia [23–25]. This is might due to differences in culture, sociodemographic, and difference in living status.

The lifetime prevalence of sexual violence among female night students in this study was 49.1%. This finding is similar to studies conducted in Wolaita Sodo University students 45.4% [26]. This could be because young people are unaware of their sexual rights, and the nighttime increases the likelihood of violations. This finding is much higher with different studies in Ethiopia; in Debre Markos town 24.2% [15], Dilla town 13.2% [27], Butajira town 32.8% [28], Aleta Wondo town 26.3% [14], and in Harar town 25% [29].

The factors associated with gender-based violence and sexual violence were investigated in this study. Gender-based violence was associated with rural childhood residence, poor school performance, lack of discussion on sexual and reproductive health issues with family and or friends, poor family control, and sexual activity. Living with a biological partner, family support, and peer pressure were all factors associated with sexual violence.

Students who were rural childhood residence increased three times the odds of GBV as compared to urban childhood residence. This finding is supported by studies from Southeast Nigeria, Nigeria, and Awassa University in Ethiopia [30–32]. This could be due to limited access to information and knowledge about reproductive health issues; the burden of violence against women, girls, and children; and access to health care in rural areas. Students who had poor school performance increased three times the odds of GBV as compared to students who had good school performance. Similar findings were reported in Debre Markos and Mekelle town in Ethiopia, and in the University of Ibadan, Nigeria [15, 33, 34]. Students who were being sexually active increased almost four times the odds of GBV as compared to their counterparts. This finding corresponds with a study from Wolaita Sodo, Aleta Wondo town, and Dilla town, Gedeo zone [14, 16, 27].

Lack of experience of discussion about sexual and reproductive health issues with their family and or friends has increased almost four times the odds of GBV as compared to their counterparts. This finding was a consistent study at Wolaita Sodo University that showed that discussions of sexual issues with her partner reduced 74% of the risks of sexual violence [16]. The fact that discussion of sexual and reproductive health issues between parents and adolescents is one of the strategies that enable adolescents to delay sexual debut or avoid sexual and physical violence. Adolescents should learn the human reproductive system and problems related to the sexual and reproductive issue and appropriate family responsibility.

Living with a biological partner is reducing the risks of sexual violence by 72%. This finding is similar to studies in Harar town, Eastern Ethiopia, Northeastern Nigeria, Uganda, and [29, 35, 36]. Family support significantly reduced sexual violence. This finding is consistent with a study in Butajira town, Ethiopia [28], and studies in Sweden and Australia [37, 38]. This is might be due to the fact that family support in case of income, physical control/supervision, and encouraging concerning on educations leads to avoid exposure for sexual and physical violence. Peer pressure is significantly associated with sexual violence. This finding is consistent with the studies in Harar town, eastern Ethiopia, and World Health Organization (WHO) reports [29, 39]. The supporting study states that adolescence is the time when a person is most susceptible to peer pressure because peers become an important influence on behavior during adolescence, and peers are engaging in similar behavior [40, 41].

## 5. Conclusion

In the study area, the lifetime prevalence of gender-based violence was high. Gender-based violence has been identified as a prevalent issue among female night students. The lifetime prevalence of sexual violence, physical violence, and emotional violence was 49.1%, 57.5%, and 41.6%, respectively. Factors associated with gender-based violence included rural childhood residence, low school performance, lack of experience in sexual and reproductive health conversations, poor family control, and being sexually active. The risk factors for sexual violence were young people living with their parents, peer pressure, and family support. This finding suggests that the primary prevention of gender-based violence is urgently needed in elementary and high schools. The Ethiopian government and other stakeholders should focus on reducing the risk of gender-based violence at the school level and provide regular health promotion on sexual and reproductive issues through school clubs.

## Figures and Tables

**Table 1:** Sociodemographic characteristic of female night students in public institution of Bahir Dar City, Amhara Region, Ethiopia, 2019.

Variables	Frequency	Percentage
Age		
15-19	452	57.4
20-24	336	42.6
Religion		
Orthodox	683	86.7
Muslim	64	8.1
Others ^∗^	41	5.2
Ethnicity		
Amhara	726	92.1
Others ^∗∗^	62	7.9
Educational level		
Grade 1-8	514	65.2
Grade 9-10	274	34.8
Living condition		
Alone	118	15
With family	214	27.2
With husband/boyfriends	90	11.4
With female friends	121	15.4
With employer	245	31
Marital status		
Married	128	16.2
Had boyfriend	237	30.1
Single	423	53.7
Monthly pocket money		
<2000	324	41
2000-4000	280	35.6
≥4000	184	23.4

^∗^Others include Protestant and Catholic. ^∗∗^Others include Tigray and Oromo.

**Table 2:** Sexual characteristic of female night students in public institution of Bahir Dar city, Amhara Region, Ethiopia, 2019.

Variables	Frequency	Percentage
Ever had sexual intercourse		
Yes	490	62.2
No	298	37.8
Age at first sex (*n* = 490)		
10-16	81	16.5
17-19	319	65.1
≥20	90	18.4
Number of sexual partner in life (*n* = 490)		
One	180	36.7
Two and above	310	63.3
How you exposed to first sexual intercourse (*n* = 490)		
With marriage	67	13.7
Self-interest	50	10.2
Peer pressure	220	46.7
Forced	59	12
For money	94	19.2
Discussion SRH issue with family and or friends (788)		
Yes	227	28.8
No	561	71.2
Have constant sexual partner (*n* = 490)		
Yes	202	41.2
No	288	58.8
Lifetime sexual violence (*n* = 788)		
Yes	387	49.1
No	401	50.9
Sexual harassment (*n* = 788)		
Yes	514	65.2
No	274	34.8

**Table 3:** Consequence of gender-based violence among female night students in a public institution of Bahir Dar city, Amhara Region, Ethiopia, 2019.

Variables (*n* = 490)	Frequency	Percentage
Unwanted pregnancy	72	14.7
Abortion	61	12.4
Sexually transmitted infection	132	27
School dropout	152	31
Depression	323	66
Anxiety	303	62
Self-blame	216	44
Rejection from family	44	9
Rejection from friends	29	6
Alcohol dependency	17	3.4
Sexual dependency	33	6.7
Injury around the genitalia	201	41

**Table 4:** Factors associated with gender-based violence among female night students in Bahir Dar city, Amhara region, Ethiopia, 2019.

Variables		Gender-based violence		COR (95% CI)	AOR (95% CI)	*p* value
		Yes	No			
Childhood residence	Urban	283	180	1	1	
	Rural	277	48	3.67 (2.56-5.26)	3.37 (2.17-5.54)	0.001
School performance	Good	93	92	1	1	
	Poor	467	136	3.40 (2.40-4.80)	3.44 (2.13-5.56)	0.001
Discussion SRH issue with family and/or peers	Yes	90	137	1	1	
	No	470	91	7.86 (5.55-11.14)	3.68 (2.07-6.54)	0.001
Family control	Yes	181	184	1	1	
	No	379	44	8.76 (6.03-12.72)	5.62 (3.25-9.71)	0.001
Being sexually active	Yes	388	132	1.64 (1.19-2.26)	3.79 (2.35-6.12)	0.001
	No	172	96	1	1	

**Table 5:** Factors associated with sexual violence among night female students in Bahir Dar City, Amhara Region, Ethiopia, 2019.

Variables		Sexual violence		COR (95% CI)	AOR (95% CI)	*p* value
		Yes	No			
Living with biological partner	Yes	64	168	0.27 (0.19-0.38)	0.28 (0.19-0.41)	0.001
	No	323	233	1	1	
Peer pressure	Yes	229	85	5.40 (3.94-7.37)	5.73 (4.11-7.98)	0.001
	No	158	316	1	1	
Family support	Yes	123	239	0.32 (0.24-0.42)	0.31 (0.22-0.43)	0.001
	No	264	162		1	

## Data Availability

The data used to support the findings of this study are available from the corresponding author upon formal request.

## Abbreviations

GBV: Gender-based violence
EDHS: Ethiopia Demographic and Health Survey
WHO: World Health Organization
AOR: Adjusted odds ratio
COR: Crude odds ratio
SV: Sexual violence.
